# Community as Locus for Health Formal and Non-Formal Education: The Significance of Ecological and Collaborative Research for Promoting Health Literacy

**DOI:** 10.3389/fpubh.2014.00283

**Published:** 2014-12-22

**Authors:** Sofia C. Pais, Mariana Rodrigues, Isabel Menezes

**Affiliations:** ^1^Centro de Estudos Sociais, Universidade de Coimbra, Coimbra, Portugal; ^2^Centro de Investigação e Intervenção Educativas, Faculdade de Psicologia e de Ciências da Educação, Universidade do Porto, Porto, Portugal

**Keywords:** health literacy, methodological pluralism, collaborative approaches, children and adolescents, formal and non-formal education

## Abstract

The World Health Organization (2002) considers that a balance between government, community, and individual action is necessary for health education and promotion, recognizing that non-governmental organizations, local groups, and community institutions are central in this process. This argument reinforces the idea that individuals should be empowered and encouraged to make use of accurate health-related information. This paper highlights the potential of a socio-political perspective for the development of health literacy within children and adolescents and presents two studies conducted in two daily life contexts: a community organization and a school. Both studies are based on methodological pluralism and collaborative research approaches and explore the promotion of health knowledge in formal and informal settings. Study 1 is based on a mixed methodology, using focus group discussions and questionnaires with children and youth with chronic diseases to explore the perceived impact of their participation in support associations. Study 2 presents four intensive case-studies in schools where adolescents used community profiling, a participatory research methodology, to explore health rights and access to healthcare in both a historical and prospective vision. The results enable a deeper understanding on how powerful tool ccommunity resources can be for individual and collective empowerment on health issues.

## Introduction

During the last decades, the concept of primary health care has made significant progress throughout the world: today people have an inalienable right to health and health care without prejudice with regard to gender, age, religion, ethnic grouping, social class, material circumstances, political affiliation, or sexual orientation (1). The contribution of the World Health Organization (WHO) with the Health-For-All 2000 strategy, originally formulated in Alma-Ata (Kazakhstan) in 1978, served as an inspiration for all countries. The intention was to emphasize the importance of primary health care and, moreover, the promotion of health for all people (2). Recent studies, such as Walley and colleagues (3), revisited the declaration of Alma-Ata highlighting the importance of powerful messages in the human rights field (4). If, on the one hand, health has to involve something more than the absence of illness or disease and it is considered as a fundamental human right, on the other hand, the declaration of Alma-Ata advanced the premise that health promotion should be understood as a core social objective, which crosses the boundaries of the state and the health sector (5) – and is therefore a goal shared by diverse social institutions. Since then, other international conventions such as the Ottawa Charter, the Copenhagen Declaration and the Habitat Agenda corroborate the importance of (re)meeting health and health care environments as rights, stressing the need to reduce inequalities determined by social conditions (6).

In parallel to these statements of access to health as a human right, the scope of the conception of health has also expanded to reflect a range of factors (7, 8). The WHO classification is based on an understanding of health as a “complete physical, mental, and social state, and not merely the absence of disease or infirmity” (9). Such a broad framework implies that health is more than a biomedical phenomenon (10–13), and includes social and political dimensions. In this paper, we advocate the potential of a socio-political perspective to consider both the experience of living with a chronic disease and the development of public health literacy. Such a perspective advocates that people, whether “healthy” or “ill,” are complete human beings as well as citizens with rights as well as needs (14). This implies an extension of the rights model of health that goes beyond the dichotomy between health and disease – health is closely related to its opposite, as proposed by Susan Sontag (15) they can be described as two inseparable kingdoms – as “a way of getting us all to think about the things we have in common and the barriers we all face” [Ref. (16), p. 11], contrary to reinforcing individual conditions, limitations, and needs. In this sense, health rights and health promotion are not exclusive concerns of biomedical professionals and settings, and involve other experts and territories (8). In fact, exploring health through an ecological perspective, as suggested by Bronfenbrenner (17, 18), involves taking into account the inter-connections between multiple contexts in the individual’s life experiences, recognizing that different actors and a range of environments play an essential role in people’s lives, especially for those with particular health/disease condition. On one hand, “an ecological perspective draws attention to how individuals with diverse skills, resources, and worldviews cope with and adapt to their local community contexts” [Ref. (19), p. 400]; on the other hand, these community settings with their own specificities and dynamics naturally work to provide opportunities to learn and experience numerous situations (14) – community resources can be a powerful tool for individual and collective empowerment, as there are cultural factors, economic dynamics and social structures whose contribution is vital for the improvement of people’s quality of life (20, 21).

## Methods: Methodological Pluralism and Collaborative Approaches in Research

In recent decades, a number of authors have recognized that traditional scientific approaches are not the only possibilities for social research and scientific knowledge production (22–24). Particularly since the 1990s, publications in the field of educational, psychological, social, and community intervention started to discuss the potential of a mixed methods approach and advocating its use combining quantitative and qualitative methods – based on the strategy of fitting the method to the research question (and not the reverse) (22). It is currently widely accepted that quantitative and/or experimental approaches can be complemented with qualitative alternatives, such as narratives and other post-modern methods, ensuring an increase in the accuracy and consistency of the research process [Ref. (25), p. 202]. Non-traditional research approaches can generate knowledge that is similarly valid and, from the point of view of methodological pluralism, all scientific approaches have their place and are to be valued (25) – methodological pluralism allows for a more comprehensive and complex understanding of social phenomena (26). In truth, there is no method that has only advantages or disadvantages: “No single approach to research is best overall, rather, what is important is that the methods be appropriate for the questions under investigation. No single research method is inherently superior to any other” [Ref. (25), p. 245].

Together with this trend toward pluralism in methods, there has also been support for the use of collaborative research (19, 27–29), emphasizing the active involvement of participants in the research process, especially in “natural” community settings. It is argued that “a collaborative relationship must be developed, a ‘doing with’ as opposed to ‘doing to’ the community” [Ref. (27), p. 30]. In this sense, involving local actors in the identification of research topics and significant informants, in the collection and analysis of data, and in the discussion and presentation of the results is a golden key for enhancing the ecological and catalytic validity of the research (14, 30). As Trickett and Espino (28) stress “developing research in real-world settings on real-world problems and policies through learning collaborations between scholars and citizens” (p. 13) is an essential component of community research. Kelly (27) argues that “a benefit of the ecological approach is that practical help is derived from an intimate familiarity with the social processes of the network” (p. 588). In this sense, “potentially collaboration integrates the scientific goal of ecological validity and the action goal of citizen empowerment” [Ref. (27), p. 13].

The potential of such as approach to the promotion of public health literacy and health education lies in the fact that collaborative perspectives promote the co-production of participant knowledge within the research itself (14, 27, 28, 31). Mobilizing community resources and actors in a collaborative research process implies “understanding how the diversity of settings in which people actually do live well, operate” [Ref. (14), p. 19], deeply exploring and considering the meanings people attribute to health and disease, taking explicitly into account and balancing power relationships between “researchers” and “community members” (32), and increasing the potential of research results to generate sustainable community and individual changes if the process involves and is intelligible for participants. Corroborating Hawe’s (33) argument that “the job of the health promotion intervention is to harness and enhance the natural problem-solving and helping processes in the community” (p. 201). Health education and community health have seen an increasing use of collaborative approaches (34, 35) reflecting “the assumption that health problems have multiple determinants, including those over which individuals have little or no control; that community-level change necessitates community-level involvement; and that the success of community involvement hinges on community capacity to mobilize effectively” [Ref. (28), p. 12].

In this paper, we will present two different studies carried out in 2010 and 2012 with Portuguese children and youth in two daily life contexts: a community organization and a school. Both studies share a socio-political and ecological perspective on health and disease, and use methodological pluralism and collaborative research to explore the experience of living with a chronic disease and the development of public health literacy. In our view, ecologically based research and the use of participatory approaches can contribute significantly to health research projects (25) and it is this dimension that we intend to emphasize across the paper. In both cases, informed consent, confidentiality, and anonymity were assured to all the participants, and parents gave written consent to their children’s involvement in the research (36, 37), according to the ethical standards of the scientific board of the Faculty of Psychology and Education Sciences of the University of Porto. A common goal was “benefiting the participants either through direct intervention or by using the results to inform action for change” [Ref. (38), p. 175]. We will present the characteristics, methods, and results for each study, and follow this with a joint discussion of their main contributions for the promotion of public health literacy and health education.

## Study 1: Community Associations as Partners in the Well-Being of Children and Adolescents with Chronic Diseases

A chronic disease is a “disease of long duration and generally slow progression” most often associated to non-communicable diseases (29). Symptoms of pain, depression, fatigue, and the tendency to lower persistence in tasks might be some of the effects triggered by the disease, the medication and/or by absences caused by hospital attendance (39, 40). Expenses with medication therapy, hospital visits, and changing family routines are some other factors requiring sometimes difficult adjustments (41, 42). In this sense, community organizations or support associations whose activity is to defend the quality of life of people with chronic disease can play a significant role as health education contexts. The literature illustrates the benefits of involvement in these community organizations, especially those with a more informal nature (43), such as emotional support, sharing experiences, and acquiring new information. As Kelly (27) argues, “through participation in a network people learn that there are other who share their values, [articulating] their common struggles and working for a collective acknowledgment of shared goals” (p. 585) – claiming rights and advocating for better life conditions are significant dimensions of these community contexts (44, 45). Similarly, these support associations tend to be seen as promoters of active citizenship by contributing to empowerment (27, 45).

The main goal of this study is to understand how these community organizations contribute to the lives of children and adolescents with chronic diseases, considering the acquisition and improvement of health literacy and the promotion of the well-being and empowerment. Support associations are conceived as non-formal health education contexts, where children and adolescents can acquire relevant information regarding their own health condition, share strategies to cope with the disease and with the physical and attitudinal barriers they face in their daily life, and develop a critical consciousness regarding their health rights and their role as citizens in a community. The study uses a mixed-methodology design with an emphasis on collaborative approach toward participants.

### Methods and settings

This study is part of wider research on the role of support associations in the promotion of the well-being of children and young people with chronic diseases (asthma, arthritis, diabetes, epilepsy, and heart and respiratory diseases) and their families. Assuming that methodological pluralism may allow for a more comprehensive and complex understanding (26) of this phenomena, we used a two-phase design with focus group discussions and questionnaires. Data were collected in 2010 and 2011 and participants were recruited through support associations and hospitals.

During phase 1 we used focus groups to explore, on one the hand, the general experiences of children and young people with chronic disease (diabetes *mellitus*) in their life contexts and, on the other hand, their perceptions of the benefits of involvement in support associations. In this paper, we will refer to two focus group discussions: one with three children (two boys and one girl), with diabetes, aged between 8 and 12, with and without involvement in support associations; another with three members and leaders of support associations, all of them boys with diabetes, aged between 19 and 24 years old. Even if the number of participants was smaller than typical focus groups, the interactive nature of the discussion was fully accomplished certainly due to the participants’ engagement with the topic. The focus group occurred in contexts familiar to the participants (home, school). In all cases, confidentiality and anonymity were assured, and the participation of children was approved by their parents. As Miller and Shinn (46) argue it is possible to learn a lot from the ordinary knowledge, skill, and craft of people – the focus group with the children was very important for gaining a full understanding of living with chronic disease, but the focus groups with members and leaders of support associations was also important to understand the role of associations as non-formal health education contexts. This methodological option allowed, on one the hand, a privileged access to a shared knowledge among group members about their vision of chronic diseases (in this specific case, diabetes *mellitus*) and, on the other hand, explored an active involvement of children and young people in the discussion of their life and health conditions. The data analysis was based on content analysis (47). All names mentioned below are fictional.

The results of the focus groups helped us identify dimensions that could be explored in a quantitative study in phase 2. The self-report questionnaire on the effects of support associations on children and adolescents’ quality of life was administered to children and adolescents (older than 12) (*n* = 176), and took about 20 min to complete. The questionnaire included several scales adapted from other instruments; all items were scored on five points Likert-type scales, from 1 (strongly disagree) to 5 (strongly agree) or from 1 (not frequently) to 5 (very frequently). Data analysis was performed using the Statistical Package for the Social Sciences (SPSS), version 18, and Analysis of Moment Structures (AMOS) software. Confirmatory factorial analysis was conducted in order to test the dimensionality of the questionnaire and the adjustment of the data to the theoretical models. The scales include the following dimensions: (a) Well-being [χ^2^(43) = 65.54, *p* < 0.015; CFI = 0.95; RMSEA = 0.053] adapted from the European Social Survey (48) scales, with two dimensions: social well-being (“I feel that people respect my illness”) and personal well-being (“I feel I am free to decide for myself how to live my life and conduct my health”); (b) Satisfaction with physical health [χ^2^(8) = 10.03, *p* < 0.262; CFI = 0.96; RMSEA = 0.037] adapted from the WHO Quality of Life-BREF (WHOQOL-BREF) (49) and Quality of Life Index (50) (“I need to take medication to carry out my daily activities”); (c) Satisfaction with life contexts [χ^2^(9) = 18.93, *p* < 0.026; CFI = 0.97; RMSEA = 0.077] adapted from WHOQOL-BREF, OMS, 2004 and the Quality of Life Index (50) (“The hospital appointments are scheduled for times when I do not have classes or tests”); (d) School integration [χ^2^(8) = 9.44, *p* < 0.276; CFI = 0.99; RMSEA = 0.035] adapted from the International Classification of Functioning, Disability and Health (ICF) (51) (“I have always been able to do activities in order to maintain my participation in school”); and (e) Empowerment [χ^2^(32) = 48.88, *p* < 0.029; CFI = 0.94; RMSEA = 0.053] a scale constructed for this study (52) including three dimensions: interactional empowerment (“When it is necessary, I take the initiative to look for people, services, and/or institutions to help me find solutions to my problems”; “Health policies have better results if people with a chronic illness are involved in them”); self-centered behavioral empowerment (“I can choose another health professional to follow my health”; “I played an active part in decisions related to my health”); and other-centered behavioral empowerment (“I helped other people to overcome problematic situations related to their illness”; “I got other people to hear my opinion on issues related to chronic diseases”).

### Results

Results from the focus groups show that children appear to be cautious in managing their relationship with their peers when it comes to sharing their health condition – the dichotomy between the visibility and invisibility of a chronic disease, in this case diabetes, suggests that these children rethink strategies for fostering integration into groups. Their dialog reinforces how powerful this invisibility might be and peers’ indifference for their social inclusion, as well as the pressure to “be equal” to other children:

“Ted (11 years) – … *my friends don’t know*.

John (12 years) *– Mine think it’s cool when I’m pricking my finger*.

Maria (8 years) *– When I present myself to a new friend in class I generally say I’m diabetic*.

Ted – *not me* … *when I know a friend I wait he’ll be a real friend and then I say I have diabetes*.

John *– [when they ask me anything about diabetes] it’s a sign they are interested in meeting me*.

Ted – *No, we don’t talk about it [diabetes]*.”

Similarly, young members and leaders of support associations recognize that some families are “afraid of the social stigma” (Peter, 19 years), and support associations can help people gain more autonomy and power through self-knowledge. As Sam (20 years) said “knowledge is power, so the best way to be different is to know the tricks you can do [referring to take the advantage of pricking the finger in several ways]”. They all agreed that sometimes:

“Peter – *the problem is not so much the people who are around you, but our tendency to give them a wrong idea*.

Sam – *We shouldn’t be seen as poor or miserable people!*

Adam (24 years) *– We might not be equal to our friends but we can do everything they do*.”

Therefore, they seem to assume that their rights are ensured if their specific needs were taken into consideration (53), making life with a chronic disease a citizenship question (54) more than a simple biomedical condition. This also emerges when they discuss the problems that arise in the interaction between school and hospital. Children comment on how their daily life is made of individual arrangements – as Ted explains what happens when he has a doctor appointment at the same time as a school test: “Usually the other class is doing a test the next day, so I try to do it at that time. Or I go to the next room or I am in a corner in the same room” – instead of generating an institutional and collective response that recognize that people with chronic diseases have rights that should imply a shared responsibility connecting institutions, communities, and political structures, as Peter claims.

Finally, the role of associations clearly depends on the age and experience of participants. Even if children are not very familiar with support associations, two of them already participated in leisure events they view as important because “it helps people live with diabetes, and thus relax” (Maria). Informal networking was also emphasized by Peter who considered participation fostered “the motivation and consequent sociability of people with chronic illness” and by Sam who considered “the emotional balance and sharing of experiences” was instrumental for adaptation and well-being. But associations can also facilitate access to privileged knowledge about therapeutic innovations and “the update for existing therapies” (Adam) thus promoting the health literacy of young people with chronic diseases.

**Figure 1:**
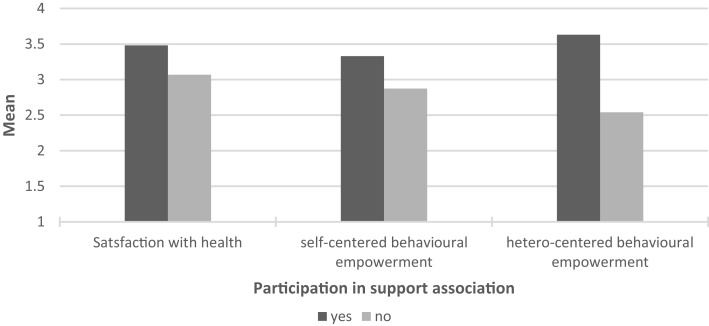
**Significant mean differences between participants and non-participants in support organizations**.

The results from the quantitative phase allowed us to explore further the role of involvement in support associations in the various dimensions of empowerment and satisfaction with health. Considering the impact of getting involved in support associations or community organizations, Multivariate tests [*F*(8, 153) = 5.833, *p* < 0.001] reveal significant differences with advantages for the young participants in terms of satisfaction with health [*F*(1, 160) = 15.347, *p* < 0.001] and self [*F*(1, 160) = 7.501, *p* = 0.007] and hetero-centered [*F*(1, 160) = 37.003, *p* < 0.001] behavioral empowerment; there are no significant differences for well-being, satisfaction with life contexts and school integration. Thus associative involvement seems to have a significant impact on relevant dimensions of the quality of life of children and adolescents with chronic disease: participants do feel more satisfied with their health condition, more capable of making decisions regarding their own health and to influence other people regarding issues related to chronic diseases (see Figure 1).

It is important to recognize that, when we look at the most relevant predictors of well-being and empowerment in children and adolescents with chronic disease, the significance of community daily contexts, such as the school and healthcare services is apparent, as school integration predicts both empowerment and well-being and satisfaction with life contexts, including access to healthcare, predicts interactional empowerment, and well-being (12). Therefore, school integration and satisfaction with life contexts such as hospital and other healthcare facilities appear to play an essential role in the well-being and empowerment of children and adolescents with chronic disease. This reveals, on one side, how relevant are the institutional and collective responses to people’s needs in order to guarantee their rights (53) and, on the other hand, it reinforces what Rappaport [Ref. (14), p. 19] defines as the central role of “natural support systems”.

## Study 2: Community Profiling on Health Rights as a Tool for Participatory Citizenship and Health Literacy

Community profiling (CP) can be defined as a participatory and collaborative research methodology based on a process of describing the needs and resources of a particular community, considering various profiles: e.g., territorial, demographic, economic, services, institutional, anthropological, psychological, and future (55). This process requires the “active involvement of the community itself” [Ref. (56), p. 5] in the formulation of the priorities for community intervention and action. According to Menezes (30), it is important to diversify both the sources of information and the data collection methods, to ensure the inclusion of diverse perspectives. CP has been found effective in improving community development and the quality of life of community members (12, 57–61), by promoting citizens’ participation in local programs (e.g., health, environment, local social audits), as well as the creation of networks among citizens and existing governmental and non-governmental organizations, in order to identify specific weaknesses and strengths of their community (62). In the domain of public health, CP can have an added value by strengthening relations between those who use healthcare services and those who provide them. Therefore, it is relevant to engage communities in health improvement for many reasons, including the determination of local healthcare needs and aspirations, the promotion of health and reduction of health inequalities, the enhancement of health service design and the quality of care, and the strengthening of local accountability [Ref. (63), p. 3].

More recently, CP has been used in the field of education as a strategy for learning citizenship as young people can interact with service users and other members of the community (local agencies and organizations) in research projects to identify needs and to evaluate existing resources (64). It is essential to understand that citizenship education is not confined to the school context, as young people are in an ongoing learning process with the situations, practices, relationships, and experiences that compose their daily lives (65–67). CP is a methodology that encourages young people to progressively take more responsibility in selecting, planning, and leading activities that address topics of their own interest and provide opportunities for interaction with relevant others (e.g., general population, professionals, stakeholders, political representatives…) (68). These kinds of learning experiences can have a positive impact on various aspects of personal and social development, namely young people’s ability to challenge assumptions about their community, research and teamwork skills, and capacity to apply academic knowledge to their everyday life, thus enhancing their literacy in various fields [Ref. (64), pp. 786–787]. Therefore, the implementation of CP in the school context can facilitate the empowerment of participants (pupils, teachers, parents…), as it favors a flexible and creative approach in a participative, collaborative, and negotiated research process [Ref. (30), p. 60]. Moreover, it can constitute an opportunity to gain a greater understanding of everyday health and healthcare needs of a particular community, and subsequently contribute to health needs assessment and improvement processes [Ref. (69), p. 56].

In the present study, CP was used as a collaborative research tool for participatory citizenship and health education, involving pupils and teachers from various public schools. Given that Portugal has a recent democratic history – only in 1974 a military revolution restored democracy after a period of dictatorship that lasted for 48 years – pupils selected one of various possible topics (social rights, civil rights, political rights, health rights…) and implemented a community-based research to develop three profiles: past (before the democratic transition of 1974), present, and future (in 35 years). This paper discusses both the implementation and results of CP for those groups involved in researching about health rights and healthcare needs and resources.

### Methods and settings

The present study involved four intensive and comparative case-studies within state schools (ranging from primary to secondary level), between January and June of 2012, with more than 100 children and young people organized into 25 workgroups [see Ref. (70) for a more thorough description of the process]. In this paper, we will present results from two secondary schools in the city of Porto (Portugal). These schools have different historical, demographic, and architectural characteristics: school 1 is a former vocational school that used to have a high reputation, but that has lowered the pupil population in the last years and the facilities have been poorly maintained; school 2 has a reputation as high-ranking school with recently renovated facilities. In school 1, pupils came from grades 10 and 11 and were attending a vocational course; the teacher implemented CP as part of a project course; in school 2, pupils were from grade 12 and CP was developed in the course in Geography. Pupils negotiated the topics with their teachers, and three workgroups, with a total of 9 pupils, chose to study health rights (e.g., do immigrants or poor people have the same health rights?), healthcare services (e.g., is it true that there are waiting lists for medical services? How much do people have to pay when they go to a public health center or hospital (user charges)?), and healthcare needs and resources (e.g., Is there a need to increase the number of public health centers? What is the cost of drugs? Do generics really save money?).

The process was supervised by the teachers and two researchers from the University of Porto, who assisted pupils in (i) the identification of research topics and questions and (ii) the selection of key informants, data collection, and data analysis methods. Teachers and researchers also supervised the implementation of the research, and the process of data analysis. Finally, they supported pupils in the preparation of a poster to be presented in a symposium at the University of Porto, where pupils presented and discussed the whole research process in a poster session with researchers in the field of education (doctoral students).

The workgroups selected different informants within their communities: e.g., school colleagues and staff, MDs from local healthcare centers, family, and other community members. They also learned to use a variety of research methods for data collection (such as interviews or questionnaires) and data analysis: document analysis using newspapers, magazines and books, and on-line sources in the internet; statistical analysis (descriptive statistics); content analysis; and naturalistic observation (within a local healthcare center).

The implementation process was monitored and evaluated involving on site participant observation and video documentation undertaken by the two researchers, who also conducted interviews with pupils and teachers. On the whole, data from these two schools involve a total of 6 h of video, three interviews with teachers, and 19 interviews with pupils. Data were analyzed through thematic analysis (71), a qualitative analytic method that provides a flexible approach for “identifying, analyzing and reporting patterns (themes) within data” (p. 79). In all excerpts, we use fictional names.

### Results

Pupils who chose to study health rights and access to healthcare did so because they considered these topics very important and sensitive public issues. Even acknowledging the significant improvements in healthcare in the last 35 years in Portugal they are particularly concerned about the impact of the economic crisis on health public policy: “How will it be in the future? Will the State still have money to continue to support all the expenses?” (Mary, pupil, grade 11).

Community profiling projects are regarded by all pupils and teachers as meaningful learning experiences, with a positive impact not only on their educational development, as pupils, but also on their socio-political development, as citizens. Both pupils and their teachers consider that is was an opportunity to foster communication, research, teamwork, and critical skills; these experiences allowed pupils “to access information which previously they were not familiar with” (Sean, pupil, grade 12), namely in relation to the national health care system, its main strengths and frailties, leading them to identify current problems and possible solutions in their posters.

Additionally, pupils and their teachers highlighted the importance of pupils’ autonomy in choosing the research themes, sources of information and data collection and analysis methods, as expressed by one participating teacher, “it is interesting that pupils are free to choose a topic, even if we did support them in the process, they came to choose very interesting and diverse issues” (Fred, teacher, 35 years). Besides, pupils particularly stressed the challenge and motivation of doing these kinds of practical and collaborative projects, compared with their usual schoolwork: “this was not just another chore that takes a day to be done and delivered, it is a project that takes time (…), expands our training and allows us to not only know more but also to become more critical” (Diana, pupil, grade 12). Pupils consider their participation in the poster session as particularly significant as “it is important for us to present our work not only in our school, but also in other schools and to other people, because we can listen to the opinions of other people, not only from our teachers…” (Mary, pupil, grade 11). This feedback is regarded as extremely important as a basis for their motivation and improvement of their work.

Regarding their socio-political development, pupils stress their involvement, as citizens, in the identification and evaluation of the community’s strengths and weaknesses: “when we go out onto the street to ask to community, it enriches our work, because we understand what is happening out there, outside the school context” (Alice, pupil, grade 12). Therefore, these experiences allowed pupils to become “more attentive to the reality” (Fred, teacher, 35 years); furthermore, “they had the opportunity to think, to reflect and make critical judgments about different issues (…) how they were dealt with in the past, how they understand the present situation and how they foresee the future” (Allison, teacher, 53 years).

Both teachers and pupils recognize the difficulties of implementing CP projects, mainly as it opposes the current emphasis of educational policies and the highly traditional and content-focused curriculum that gives little space for implementing collaborative and research-based methods: “in terms of curricular plan, we are confined to subjects that are highly structured and oriented to the development and deepening of contents” (Allison, teacher, 53 years). But pupils also complain about the lack of cooperation from the part of community institutions: “we went to a health center and delivered a letter stating that we wanted to do a research project, we said it was urgent and so far we have not any reply. We asked permission to film and they did not let us do it, and they never answered our request to interview people [healthcare users]” (Alice, grade 12).

## Discussion

In this paper, we have addressed formal and non-formal health education for promoting health literacy and well-being under a socio-political and ecological perspective that conceives of health issues beyond an individual and biomedical view, and considers how community institutions, organizations, and networks can be a tool for individual and collective empowerment regarding health rights. We illustrate this perspective with two studies that share a mixed-methodology design and a collaborative research approach with children and youth. The results of both studies demonstrate that children and young people are interested to know more about health rights and access to healthcare not only because they relate to their individual situation but also because they recognize these are sensitive public issues.

In what concerns study 1, learning with and from the communities – in this case, community support organizations that focus on health rights of children and youth with chronic diseases – might be seen as a very relevant way to develop health literacy, empowerment, and well-being, once they reinforce local values and act more effectively in local contexts (46). Our focus group discussions illustrate that living with a chronic disease can be facilitated through the collective sharing of experiences with others with the same condition, not only through emotional support but also because it generates useful knowledge (27) – participants clearly describe how engaging in support associations helps them acquire information regarding their disease. Moreover, support associations might be extremely important in rejecting daily life oppression that does not recognize people’s autonomy and empowerment. As Nunes et al. (4) have noted “the gap between the way health rights are inscribed in national/international legal documents and conventions and the way these rights are guaranteed in everyday situations creates the ground for this type of movements and initiatives” (p. 8). Regarding the development of public health literacy this particular study also emphasizes the relevance of reconfiguring healthcare access to promote health. Results from the quantitative study not only show how participation in support associations might foster satisfaction with health and empowerment, but also how satisfaction with daily life contexts (including the school and healthcare services) is a relevant predictor of empowerment and well-being. Thus, rethinking research and interventions with children and people in vulnerable social and health situations, and involving them more actively in the whole process, might be a significant task for the future (54).

Study 2 describes the implementation of CP in schools and considers its impact as a source of learning about citizenship rights, including health rights, and of developing public health literacy. Based on intensive case-studies in state schools, the project involved the collection of data using different methods in relation to different topics, and gave pupils the opportunity to apply theoretical knowledge to the “real world” and to develop knowledge that normally is not included in the curriculum. Thus, this collaborative research strategy may constitute a useful tool in health education, empowering young people to undertake “critical citizenship in practice citizenship that is learned in practice, not guided by an activation perspective, but by an agency perspective” [Ref. (70), p. 11]. As both the monitoring of the process and the final interviews with teachers and pupils show, this resulted in a series of gains in terms of communication, research, and teamwork skills. Pupils also seem to have acquired relevant knowledge about the health rights of citizens, the national health care system, and some of the contemporary problems under public discussion (e.g., generics, user charges). However, the consideration of health rights and access to healthcare from both a historical and prospective vision appear to have given pupils the opportunity not only to have access to new knowledge regarding the situation in their country and their community, but also to critically reflect on this, involving them in healthcare needs assessment and improvement – thus enabling them to articulate the alternatives and make political judgments that are the very nature of exercising citizenship (72).

Both studies consider the promotion of health literacy through formal and non-formal health education in community contexts, namely the school and support associations. In both cases, there are also concerns with the promotion of participants’ empowerment and an emphasis on collaborative approaches to research, even if with different degrees that clearly appear especially relevant with potentially vulnerable populations. Furthermore, the two studies explore the potential of interaction in and between community contexts that, as Rappaport (14) states, are important mediating structures that stand between the large impersonal social institutions and individual alienated people, such as the family, the neighborhood, the church and the voluntary organizations: “these are the places where people live out their lives, and the more control they have over them the better” (p. 19). The findings of both studies highlight the importance of community-based resources as tools to promote health literacy and empowerment. Finally, the two studies recognize that it is important to generate multiple discourses and interpretations on health, disease and well-being that are frequently neglected in research. In particular, the views and roles of children and young people should not be overlooked (36), the results suggest that children and young people have valid points of view with regard to matters that definitely are familiar to them.

But the studies presented here have several limitations, mainly because of limited sample sizes, the use of correlational designs or the sole focus on self-report data collection methods. Although the results support our claims for the potential value of formal and non-formal health education with an ecological and collaborative focus, more research is necessary using quasi-experimental designs and combining self-report data with other methods. Still, it is expected that the experiences presented in this paper can contribute to the development of collaborative approaches in health research projects, encouraging critical reflection about its potential strengths and weaknesses when undertaken with children and adolescents. Furthermore, we hope this paper contributes to disseminating the idea of a participatory, pluralistic, and co-responsible definition of health education, where children, families, professionals, communities, and political structures play an essential role (73).

## Conflict of Interest Statement

The authors declare that the research was conducted in the absence of any commercial or financial relationships that could be construed as a potential conflict of interest.
